# Reversed Robin Hood syndrome visualized by CT perfusion

**DOI:** 10.1016/j.radcr.2021.01.047

**Published:** 2021-02-02

**Authors:** Rajiv Advani, Else Charlotte Sandset, Espen Stjernstrøm

**Affiliations:** a Stroke Unit, Department of Neurology, Oslo University Hospital, Oslo, Norway; b Neuroscience Research Group, Stavanger University Hospital, Stavanger, Norway; c The Norwegian Air Ambulance Foundation, Oslo, Norway; d Department of Radiology, Oslo University Hospital, Oslo, Norway

**Keywords:** Stroke, CT, angiography, MRI, DWI, Carotid artery stenosis

## Abstract

Reversed Robin Hood Syndrome (RRHS) was first described in 2007 as a cause of worsening neurological deficit in the setting of an acute ischemic event. RRHS is the shunting of cerebral blood flow to nonstenotic vascular territories due to impaired vasodilation bought on by hypercapnia. A 77 year old lady presented with acute onset left hemiparesis and an exacerbation of her underlying chronic obstructive pulmonary disease (COPD). CT angiography and perfusion visualized RRHS and appropriate treatment was initiated. Treatment strategies for RRHS differ considerably to those for acute ischemic stroke. Choosing the correct treatment strategy is decisive for good clinical outcome.

## Introduction

Reversed Robin Hood syndrome (RRHS) has previously been described in cases of worsening neurological deficit after an index stroke event [1]. The steal phenomenon has traditionally been visualized using transcranial ultrasound. Previously published reports have not employed the use of CT perfusion (CTP) which has now become the mainstay of ischemic penumbra diagnostics [2,3]. This brief report presents a case of RRHS as the index event in a patient with underlying chronic obstructive pulmonary disease. As described here the patient presented with acute onset stroke symptoms. RRHS however warrants a different treatment strategy if massive cerebral infarction is to be avoided.

## Case report

A 77-year-old lady presented to the emergency room with acute onset left hemiparesis.

The patient had a history of hypertension and Chronic Obstructive Pulmonary Disease (COPD) but was otherwise healthy and functionally independent. She was being treated with 2 antihypertensive medications: 5 mg amlodipine OD and 50 mg valsartan OD.

The patient had experienced a slight weakness in their left arm and left leg on the morning of admission but had not contacted the emergency services before a gradual worsening of the symptoms later that same day.

The paramedics found the patient to be awake with a left hemiparesis, left facial palsy and a mild dysarthria. The paramedics suspected an acute onset stroke, and the patient was admitted to our hospital as a stroke code.

Upon physical examination in the emergency room the patient had an National Institutes of Health Stroke Scale (NIHSS) of 11. The temperature was 36.7 °C, pulse of 90 beats per minute and blood pressure of 169/71 mm Hg. The initial prehospital oxygen saturation was 65% while on room air, subsequently increasing to 92% while breathing oxygen through a nasal cannula at a rate of 3 liters per minute. The respiratory rate was 22 breaths per minute.

An arterial blood gas obtained while the patient was receiving high-flow oxygen through a nasal cannula at a rate of 3 liters per minute showed a pH of 7.27, a partial pressure of carbon dioxide of 81 mm Hg (10.8 kPa), a partial pressure of oxygen of 60 mm Hg (8.0 kPa), a bicarbonate of 37.3 mEq per liter and a base excess of + 10.5 millimoles per liter.

A CT scan was performed including a pre- and intra- cerebral angiography. A subsequent perfusion scan was also performed. The CT examination was performed using the Siemens Somatom Definition Flash using GE Healthcare Omnipaque intravenous contrast and standardized protocols for angiography and perfusion.

Blood work including the white-cell count, differential, hemoglobin, electrolytes, high-sensitive troponin and CRP, kidney and liver function tests were all within normal limits except for a serum potassium level of 5.0 millimoles per liter.

**Fig. 1:**
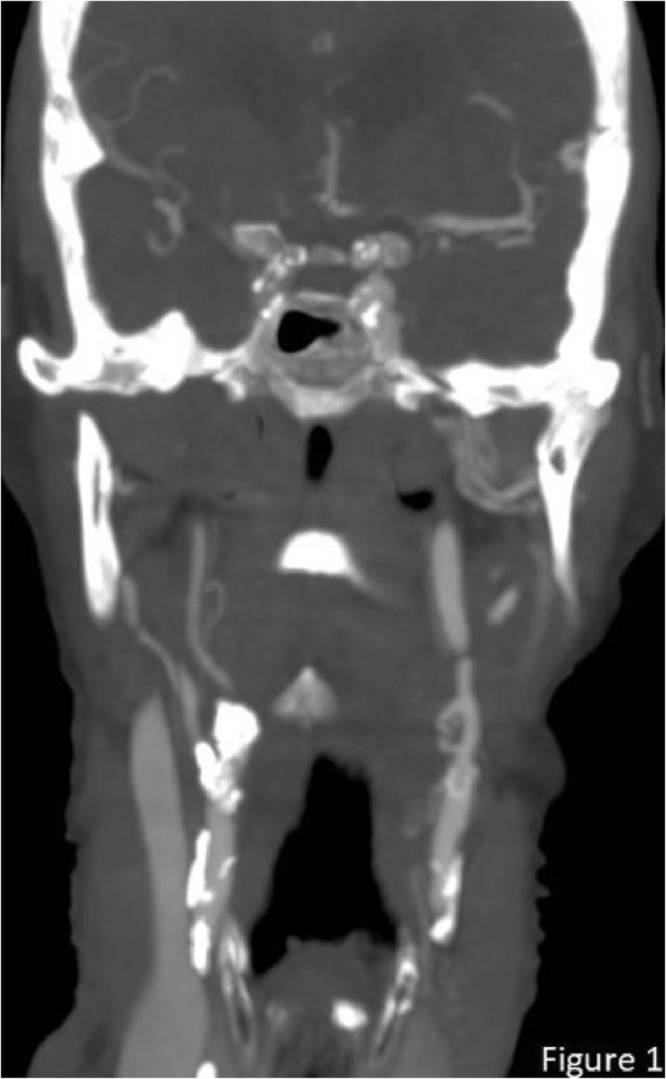
CT angiography coronal plan view showing a significant stenosis in the right internal carotid artery and the passage of contrast distal to the stenotic segment.

**Fig. 2:**
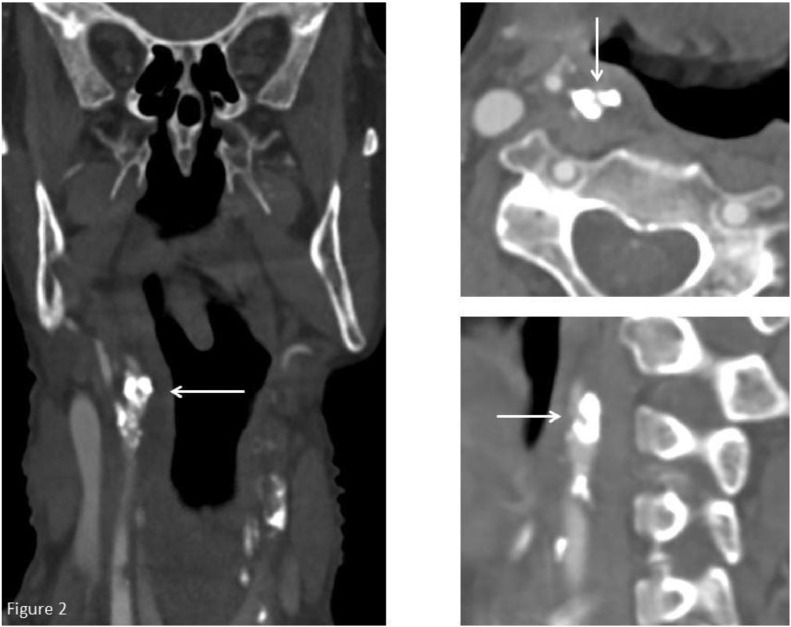
The stenosis at the origin of the right internal carotid artery shown in coronal, axial, and sagittal plan views. The white arrows indicate the stenotic segment of the artery.

**Fig. 3:**
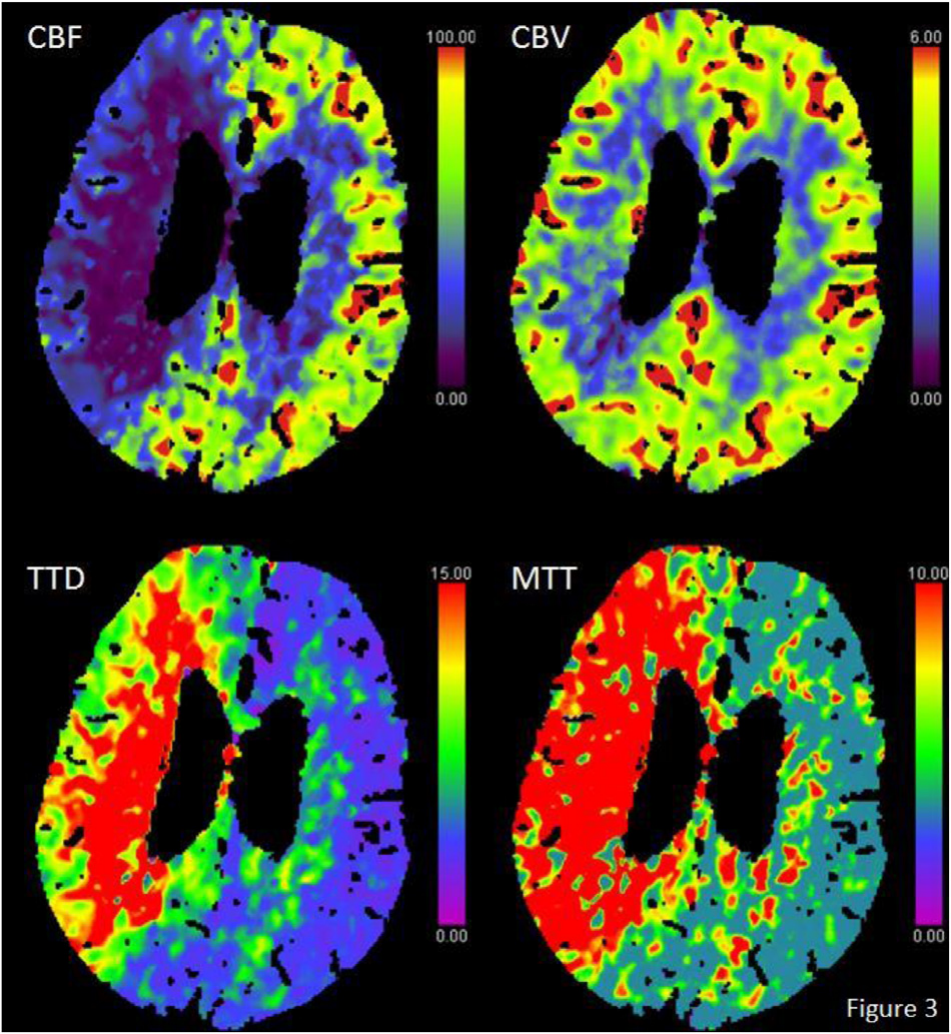
CT Perfusion axial plan view including cerebral blood flow (CBF), cerebral blood volume (CBV), mean transit time (MTT), and time To drain (TTD). CBF shows markedly decreased flow in the right cerebral hemisphere. CBV shows normal blood volume in both cerebral hemispheres. TTD shows markedly increased drain time in the right cerebral hemisphere and slightly reduced drain time in the left cerebral hemisphere. MMT shows markedly increased transit time in the right cerebral hemisphere and slightly reduced transit time in the left cerebral hemisphere.

The CT angiography showed no pre- or intracerebral occlusions. However, a 90% stenosis North American Symptomatic Carotid Endarterectomy Trial (NASCET) was seen at the origin of the right internal carotid artery (Fig. 1). This stenosis is also shown in coronal, axial and sagittal views (Fig. 2). The CTP scan (Fig. 3) showed markedly reduced cerebral blood flow (CBF) to both the anterior and middle cerebral artery territories in the right hemisphere with normal cerebral blood volume. The mean transit time and time to drain were both markedly increased. In the contralateral hemisphere the CBF is slightly elevated and both the mean transit time and time to drain noticeably reduced, reflecting an increase in cerebral perfusion.

The lack of large vessel occlusion despite the significant perfusion defects in the right cerebral hemisphere, the subtle findings in the contralateral hemisphere along with a pronounced hypercapnia on arterial blood gas were indicative of RRHS. A cerebral steal phenomenon induced by a hypercapnic state, brought on by an exacerbation of the patient's underlying Chronic Obstructive Pulmonary Disease (COPD).

## Discussion

RRHS is the paradoxical decrease in CBF to an at-risk vascular territory owing to a shunting of the flow to nonstenotic supply areas [1]. Traditionally the diagnosis of RRHS has been made using transcranial ultrasound; verifying the intracranial shunting [4]. The underlying pathophysiology is the impaired vasodilatory response in stenotic vessels leading to a shunting to areas supplied by more healthy vessels through to the path of least resistance.

In the acute setting, verification of the clinical diagnosis using the radiological tools at hand is vital. In the case described here, the lack of a large vessel occlusion can lead clinicians to strategy that cerebral perfusion should be increased by increasing systemic blood pressure. Increasing systemic blood pressure with the use of vasopressors should be avoided in RRHS as this will further exacerbate the steal thus resulting in potential hemispheric infarction.

**Fig. 4:**
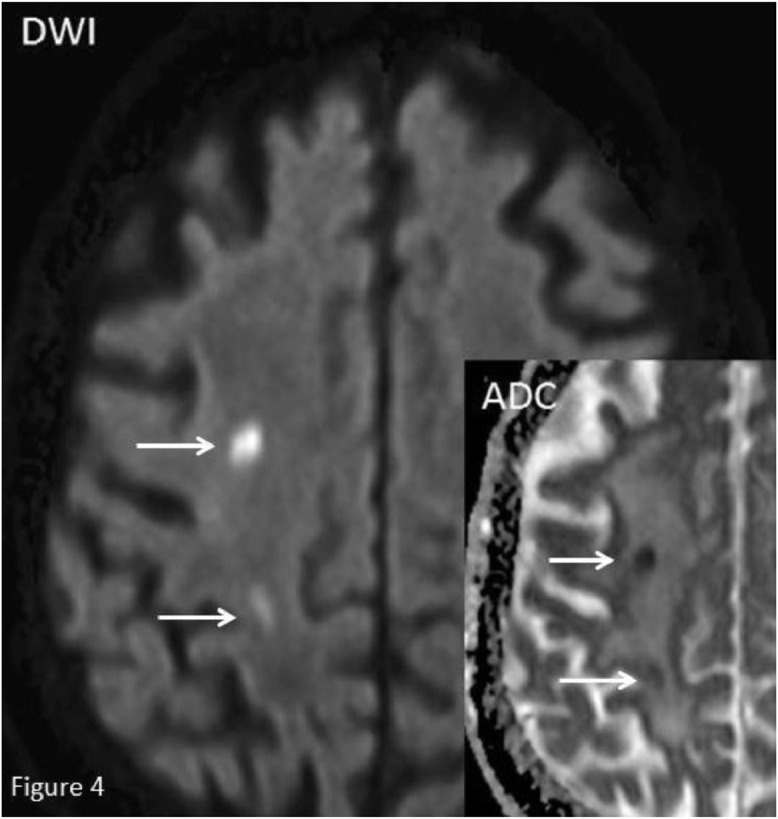
MRI Diffusion Weighted Imaging (DWI) and Applied Diffusion Coefficient (ADC) in axial plan view showing 2 small acute infarctions in the Anterior Cerebral Artery-Middle Cerebral Artery (ACA-MCA) watershed zone of the right cerebral hemisphere. The infarcts are shown using white arrows.

As demonstrated here, appropriately correcting the underlying hypercapnia should be the focus of treatment. The patient was treated for a type 2 respiratory failure using noninvasive mechanical ventilation continuous positive airway pressure (CPAP) and nebulized corticosteroids. This led to a gradual reversal of the steal phenomenon and normalization of the intracranial flow. The neurological deficits improved gradually during the subsequent 24 hours and a follow up MRI scan showed 2 small watershed infarcts in the anterior circulation (anterior cerebral artery-middle cerebral artery (ACA-MCA) watershed zone) of the right hemisphere (Fig. 4). The MRI showed no older infarctions in the right hemisphere. In cases where a radiologically significant carotid artery stenosis has led to embolic infarction in the ipsilateral hemisphere endarterectomy may be considered. Vascular surgeons were called in for a consult and the decision was made to treat the stenosis using best medical treatment. The patient was discharged 3 days after admission with an National Institutes of Health Stroke Scale (NIHSS) of 1.

The widespread use of CTP in the setting of acute stroke provides us with another method of visualizing CBF and intracranial circulation. CTP can visualize areas of decreased and increased flow relative to each other. This gives us the possibility to not only evaluate cerebral hypoperfusion and penumbra in acute ischemic stroke but also areas of hyperperfused tissue [5,6]. CTP findings should always be carefully evaluated where an occlusion isn't found and conditions such as epileptic hemiparesis, encephalitis and intracranial shunting should be considered.

The diagnosis of RRHS has previously been put forward in the setting of worsening neurological deficit shortly after an index stroke [1]. In this case RRHS was the presenting diagnosis without a preceding index stroke event. The recognition of RRHS and its underlying pathophysiology is crucial in order to determine the correct course of treatment.

Access to transcranial ultrasound is limited at many acute treatment centers and other diagnostic modalities should therefore be considered. The case presented here highlights CTP as an underutilized tool in the evaluation of intracranial blood flow.

## Patient consent

The patient consented to the publication of this brief report.
